# Metabolic Alterations Related to Glioma Grading Based on Metabolomics and Lipidomics Analyses

**DOI:** 10.3390/metabo10120478

**Published:** 2020-11-24

**Authors:** Di Yu, Qiuhui Xuan, Chaoqi Zhang, Chunxiu Hu, Yanli Li, Xinjie Zhao, Shasha Liu, Feifei Ren, Yi Zhang, Lina Zhou, Guowang Xu

**Affiliations:** 1 CAS Key Laboratory of Separation Science for Analytical Chemistry, Dalian Institute of Chemical Physics, Chinese Academy of Sciences, Dalian 116023, China; yudi_1808@dicp.ac.cn (D.Y.); xuanqiuhui@dicp.ac.cn (Q.X.); hucx@dicp.ac.cn (C.H.); liyanli@dicp.ac.cn (Y.L.); xj_zhao1@126.com (X.Z.); 2 University of Chinese Academy of Sciences, Beijing 100049, China; 3 Biotherapy Center and Cancer Center, The First Affiliated Hospital of Zhengzhou University, Zhengzhou 450052, China; chaoqizhang1917@163.com (C.Z.); liushasha910729@163.com (S.L.); feifeiren312@163.com (F.R.)

**Keywords:** glioma, metabolomics, lipidomics, grading, short-chain acylcarnitines, lysophosphatidylethanolamines

## Abstract

Gliomas are the most aggressive phenotypes of brain tumors and are classified into four grades according to the malignancy degree by the World Health Organization. Metabolic profiling can provide an overview of metabolic reprogramming at a specific stage of tumor initiation and development. Studies about metabolic alterations related to different grades of gliomas are helpful to understand the molecular mechanism for progression of glioma. In the current study, metabolomics and lipidomics analyses based on chromatography-mass spectrometry were performed on different grades of glioma tissues. Differential metabolites between glioma and para-tumor tissues were studied and used as the basis to explore metabolic alterations related to glioma grading. It was found that short-chain acylcarnitines were elevated, whereas lysophosphatidylethanolamines (LPEs) were decreased in high-grade gliomas. Furthermore, the gene expression of short/branched-chain acyl-coenzyme dehydrogenase (ACADSB), which is involved in fatty acid oxidation, was found down-regulated with glioma progression by analyzing related genes and pathways. In addition, LPE metabolism showed a significant difference among different grades of gliomas. These important metabolic pathways related to glioma progression may provide potential clues for further study on the mechanisms and treatment of glioma.

## 1. Introduction

Gliomas are the most common primary brain tumors in adults and are classified as I, II, III and IV, four grades by the World Health Organization [1,2,3]. Grade IV tumor is glioblastoma (GBM) with the highest degree of malignancy and the worst prognosis [4,5]. More than half of low-grade gliomas can transform into high-grade (grade III and grade IV) gliomas within 5–10 years of diagnosis, and it was hypothesized that the transformation of low-grade malignant astrocytes into GBMs is due to gene mutations [6]. Gene mutations can cause significant metabolome alterations, suggesting that tumor cells rely on specific metabolic pathways to complete biological processes [7]. For example, isocitrate dehydrogenases (IDH) mutations, the common genetic abnormalities in gliomas, could lead to accumulation of 2-hydroxyglutaric acid and changes in related metabolites. This revelation was a landmark in the field of glioma. The research studies initiated by IDH could provide potential strategies for glioma treatment and prognosis. Recently, Noorani et al. explored the role of EGFR mutants in IDH1 wild-type gliomas through whole-exome sequencing, transcriptomics and transposon mutagenesis forward genetic screening, and found new putative tumor suppressors [8]. There are many studies of this kind, starting with genes, which have contributed to the field of glioma. Metabolome is downstream of the genome and proteome, and more visually responds to the state of biological systems [9]. Reprogramming metabolome is an emerging hallmark of cancer, and it plays an important role in tumor growth and malignancy development [10]. In our study, we used metabolomics techniques, starting with metabolites, to attempt to discover upstream important metabolic genes and pathways for glioma from a new perspective.

Investigation of metabolic alterations among different grades of glioma tissues could provide insight into molecular mechanisms associated with glioma progression, and may suggest potential chances for glioma prevention, diagnosis, and treatment [11,12]. Some researchers have reported that the key metabolic alterations related to glioma grading include glutamate metabolism, choline metabolism and cysteine metabolism [13,14,15,16]. Although there are increasing reports on the metabolites related to glioma grading, the results for metabolic alterations are still equivocal. One possible reason for this is that tissue samples were not easily available leading to a small cohort. The small cohort of different grades of glioma tissues was sensitive to specific genetic changes and individual differences. Moreover, different employed methodologies and protocols might also contribute to this fact.

On the other hand, researchers have explored lipid metabolism differences during the progression of diffuse gliomas by gene clustering analysis, and have proven that lipid metabolic related genes could be used to discriminate the risk of poor prognosis [17]. This result indicates that lipid metabolism plays a crucial role in glioma progression. However, detailed lipids alterations associated with the malignancy progressing have been poorly investigated based on lipidomics platforms.

In the current study, we collected different grades of glioma tissues and paired para-tumor tissues to diminish the interference of individual differences, and studied metabolic alterations among different grades of glioma tissues based on the differential metabolites between glioma tissues and paired para-tumor tissues. Three platforms including gas chromatography-mass spectrometry (GC-MS) based metabolomics, liquid chromatography-mass spectrometry (LC-MS) based metabolomics and LC-MS-based lipidomics were performed to improve metabolite coverage. Moreover, further studies of molecular mechanisms were focused on important metabolites related to glioma grading.

## 2. Results and Discussion

**Figure 1:**
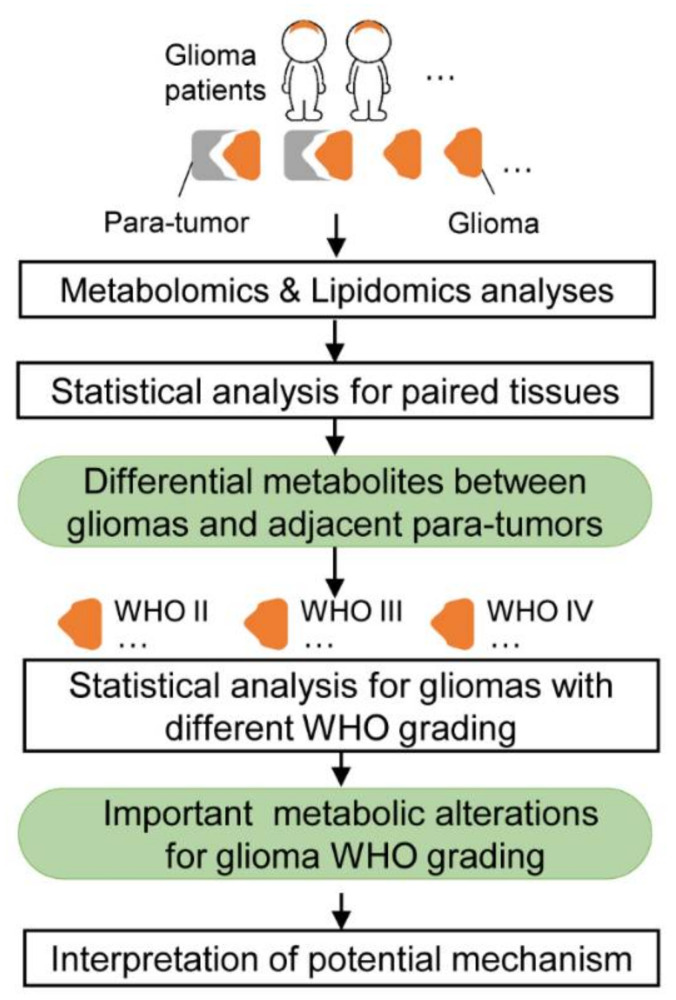
Scheme of the metabolic characterization study for different graded gliomas classified by world health organization (WHO).

To investigate the metabolic pathways and molecular mechanisms associated with glioma occurrence and progression, glioma tissues were obtained from 76 patients with different grades of gliomas, 31 of them had paired para-tumor tissues. Combining GC-MS-based and LC-MS-based metabolomics analyses with LC-MS-based lipidomics analysis, a comprehensive metabolic profile of glioma was obtained. In total, 668 metabolites were identified in glioma tissues and para-tumor tissues (Table S1), including amines, amino acids, carbohydrates, nucleotides, organic acids, glycols, carnitines, and lipids (Figure S1). Metabolic disruption of gliomas was firstly studied by comparing them with their paired para-tumor tissues to define the differential metabolites, then metabolic alterations among different grades of glioma tissues were further investigated to discover important metabolic pathways closely related to malignancy process. The workflow for this study is shown in Figure 1.

### 2.1. Differential Metabolites between Glioma and Paired Para-Tumor Tissues 

The metabolome of glioma tissues was compared with that of paired para-tumor tissues to study metabolic disruptions of gliomas. In our study, para-tumor tissues were obtained as far from the glioma tissues as the operative field permitted, which were determined by experienced neurosurgeons. Initially, Mann–Whitney U tests and Benjamini–Hochberg corrections were performed on all metabolites to discover significantly changed metabolites (*p* < 0.05 and false discovery rate (FDR) < 0.05). There were 198 detected metabolites significantly altered in glioma tissues, of which 11 metabolites were elevated and 187 metabolites decreased in glioma tissues (Table S1). Elevated metabolites included four short-chain acylcarnitines, four sphingomyelins, *N*-methyl-L-glutamic acid, adenosine, and ceramide (d18:0/16:0). Decreased metabolites were mainly lipids including phosphatidylcholines (PCs), lysophosphatidylcholines (LPCs), phosphatidylethanolamines (PEs), lysophosphatidylethanolamines (LPEs), phosphatidylserines (PSs), phosphatidylglycerols (PGs), phosphatidylinositols (PIs), fatty acids (FFAs), ceramides (Cers), sphingomyelins (SMs) and diglycerides (DGs). Lipid disruptions in gliomas have been reported. Wenchen et al. founded PC (36:1) absence in glioma tissues compared to trauma brain tissues, which is considered as a hallmark of human glioma metabolome [18]. In our study, PC (36:1) showed a lower level in glioma tissues than in para-tumor tissues (Table S1). Alan et al. reported that gray matter and white matter were characterized by increased abundance of peaks at *m/z* 834 (PS 40:6[−H]) and *m/z* 788 (PS 18:1_18:0[−H]) compared with glioma, respectively [19]. In our study, PS (18:1_18:0) also displayed a higher level in para-tumor tissues than in glioma tissues (Table S1). 

**Figure 2:**
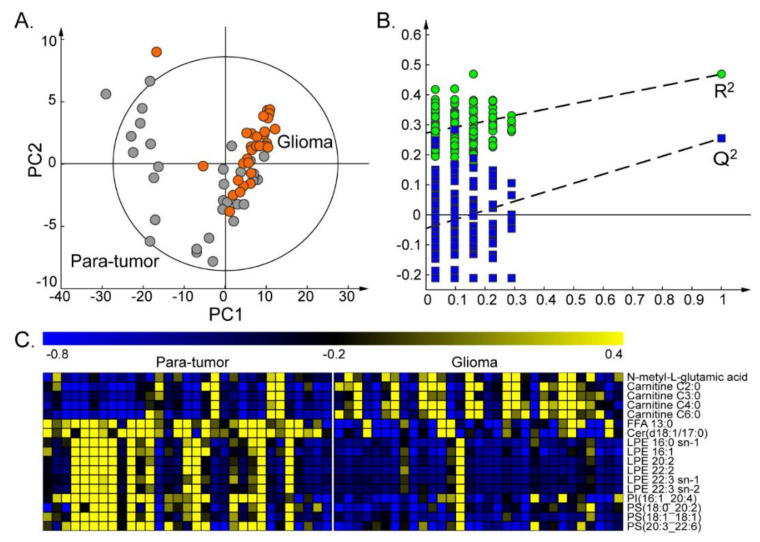
Statistical analysis for all paired glioma and para-tumor tissues. (**A**) Partial least squares discriminant analysis (PLS-DA) score plot based on all differential metabolites for gliomas (orange dots) versus para-tumor tissues (gray dots). (**B**) Model validation using a permutation test. (**C**) Heatmap showing the relative levels of the 17 important metabolites in all paired para-tumor and tumor tissues.

Partial least squares discriminant analysis (PLS-DA) was performed on those 198 differential metabolites, and a good separation trend was shown between glioma and para-tumor tissues on the score plots of the first two principal components (Figure 2A). Additionally, the model was not overfitted after being permutated 200 times for validation (Figure 2B). These 198 differential metabolites were used as a basis to discover important metabolites and pathways that may be related to glioma progression. 

### 2.2. Metabolic Alterations Related to Glioma Grading

**Figure 3:**
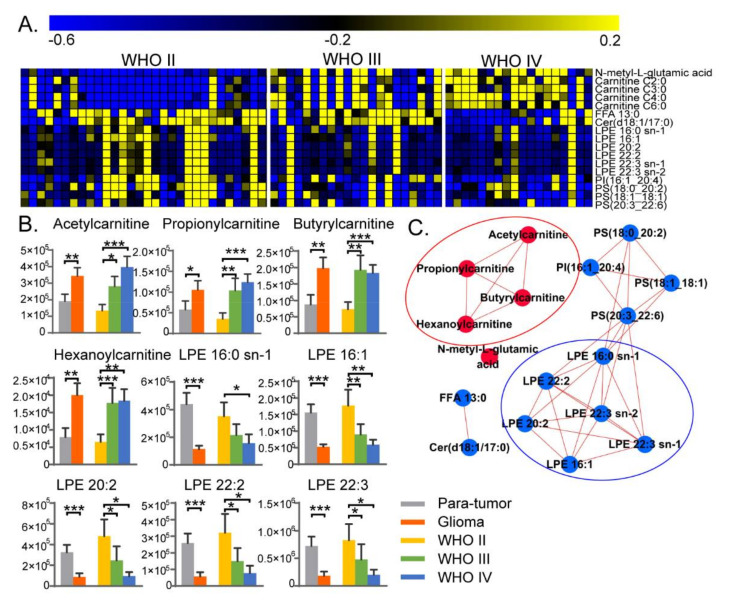
Statistical analysis for different grades of glioma tissues. (**A**) Heatmap showing the 17 important metabolites for different grades of glioma tissues. (**B**) Histogram of important metabolites related to short-chain acyl-carnitines and lysophosphatidylethanolamines (LPE). (**C**) Correlation network analysis of the 17 important metabolites for different grades of glioma tissues. Significance level, * *p* < 0.05, ** *p* < 0.01, *** *p* < 0.001.

To further investigate metabolic pathways related to glioma progression, metabolic alterations were studied among different grades of glioma tissues. In total, 69 glioma tissues were obtained from the patients and classified as grade II, grade III, or grade IV according to the WHO classification (Table 1). Mann–Whitney U tests were performed on the above 198 differential metabolites to discover important metabolites. The important grading related metabolites were chosen based on their consistent changing tendencies when comparing high-grade glioma versus low-grade glioma, glioma tissues versus para-tumor tissues. Finally, 17 important metabolites were selected (Figure 2C and Figure 3A). Among these 17 important metabolites, four acylcarnitines and *N*-methyl-glutamic acid were significantly increased both in grade III/IV compared with grade II glioma tissues (Figure 3A), and in glioma tissue compared with para-tumor tissues (Figure 2C). Meanwhile, the remaining 12 important metabolites were significantly decreased, and they were mainly lipids including LPEs, PSs and PI (Figure 2C and Figure 3A).

Amino acids and other metabolic alterations associated with glioma grading have been reported in previous studies [13,15,20], but few studies have focused on lipids. Interestingly, gene clustering analysis found that the profile of lipid metabolism-related genes showed obvious differences between low-grade and high-grade gliomas, and low-grade gliomas exhibited an enrichment in the phosphatidylinositol metabolism process [17]. This indicates that lipid metabolism is closely associated to glioma progression and may be used as a powerful prognostic biomarker. Short-chain acylcarnitines including acetyl- (C2-), propionyl- (C3-), butyryl- (C4-) and hexanoyl-carnitine (C6-CN) showed significantly higher levels in high-grade gliomas (Figure 3B). Metabolomic analysis of glioma venous and arterial blood revealed that C2-CN was absorbed by glioma, so C2-CN was the essential metabolite in glioma [21]. In the current study, acetyl-carnitine also showed a positive correlation to glioma progression (Figure 3A,B).

Next, correlation network analyses were performed on those 17 important metabolites to explore the potential relationships (Figure 3C). Short-chain acylcarnitines and LPEs had higher correlations within two sub-networks and perhaps played important roles in glioma progression, and associated pathways were further explored.

### 2.3. LPE Metabolism for Different Grades of Glioma

**Figure 4:**
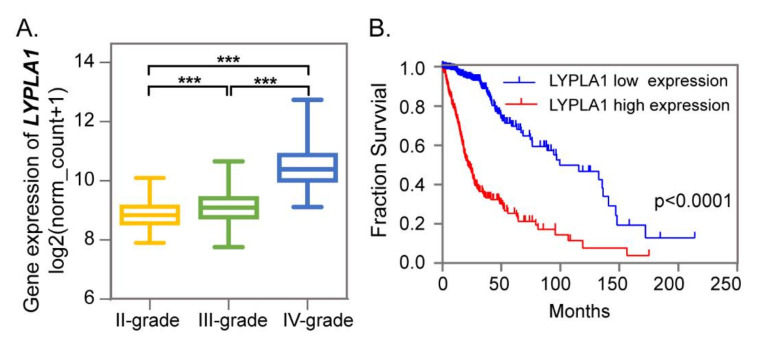
Lysophospholipase 1 (LYPLA1) gene showing higher expression in high-grade gliomas. (**A**) The gene expression of LYPLA1 in different grades of glioma tissues according to The Cancer Genome Atlas (TCGA) database. (**B**) Kaplan–Meier analysis of patients having higher or lower LYPLA1 expression levels based on TCGA database. Significance level, *** *p* < 0.001.

As described above, it was a characteristic of glioma metabolism that LPEs decreased with glioma progression. Differential LPEs showed a lower level in high-grade gliomas than in low-grade gliomas, and were also decreased in glioma tissues compared with para-tumor tissues (Figure 3A,B). Lysophospholipase 1 (LYPLA1) is the curial enzyme in LPE catabolism and catalyzes the hydrolysis of LPE to fatty acid and glycero-3-phosphoethanolamine. LYPLA1 plays a tumor-promotor role in non-small cell lung cancer cells, and is positively associated with cell proliferation, migration and invasion in vitro [22]. However, its function in the brain has not been focused on. We further explored the gene expression levels of LYPLA1 among grade II, III, and IV gliomas based on 668 glioma patients in The Cancer Genome Atlas (TCGA) database (Figure 4A). LYPLA1 gene expression increased with glioma progression and was significantly higher in grade IV gliomas. In addition, Kaplan–Meier curves showed that glioma patients with higher LYPLA1 expression had worse overall survival (Figure 4B). The higher LYPLA1 expression may lead to decreased LPE in high-grade gliomas, and shorter survivals. This indicates that the level of LPEs in glioma tissues or other biological samples may be an indicator for glioma prognosis evaluation. Further studies about LYPLA1-related molecular mechanisms would be needed, which may be valuable for glioma treatment. The importance of the phosphatidyl-lipid pathway in the brain has been proved by gene clustering analysis [17]. However, phosphatidyl-lipid metabolism is complex, and more detailed lipid alterations need to be explored in the future.

### 2.4. Short-Chain Acylcarnitines Related Metabolism for Different Grades of Glioma

As described in the Section 2.2, four short-chain acylcarnitines had higher levels in high-grade gliomas than in low-grade gliomas, and were also elevated in glioma tissues compared with para-tumor tissues (Figure 3A,B). It is well known that acylcarnitines are closely related to branched chain amino acid (BCAA) metabolism and fatty acid oxidation [23]. Carnitine palmitoyltransferases 1 and 2 (CPT 1 and CPT 2) are the initial key enzymes for long-chain fatty acid β-oxidation. CPT 1 activity could be partly reflected by the ratio of the sum of hexadecanoyl-carnitine (C16-CN) and octadecanoyl-carnitine (C18-CN) to free carnitine [(C16 + C18)/C0)], and CPT 2 activity could be reflected by the ratio of the sum of C16-CN and octadecenoyl-carnitine (C18:1-CN) to C2-CN [(C16 + C18:1)/C2)] [24]. No significant differences of the above two ratios were found for different grades of gliomas in our study (Figure S2A,B). To further explore the final products from fatty acid β-oxidation, the ratios of C2-CN to free carnitine (C2/C0) and C3-CN to C0 (C3/C0) were calculated, which can reflect the β-oxidation activity of even-numbered fatty acids and odd-numbered fatty acids, respectively [25]. As a result, C2/C0 showed no significant difference, whereas C3/C0 showed a significantly higher level in high-grade gliomas than low-grade gliomas (Figure S2C,D).

Moreover, C3-CN was a direct product of BCAA catabolism [26]. In high gliomas, the BCAA catabolism has been reported to be upregulated and to support cell proliferation in human glioma cell lines and brain tumor mouse model [27,28]. As a result, upregulated BCAA catabolism produced more C3-CN in high-grade gliomas. It has been reported that elevated C4-CN was caused by the lack of short-chain acyl-CoA dehydrogenase (ACADS) in fatty acid β-oxidation [24]. Not only elevated C4-CN, but also higher ratio of C4-CN to C3-CN (C4/C3) arose from the lack of ACADS [29]. In our study, C4-CN was elevated (Figure 3B), but C4/C3 was decreased in high-grade gliomas (Figure S2E). The elevated C4-CN may be resulted from the inhibition of ACADS; meanwhile, the reduced C4/C3 levels may be due to the more remarkably increased C3-CN from the upregulation of BCAA catabolism.

**Figure 5:**
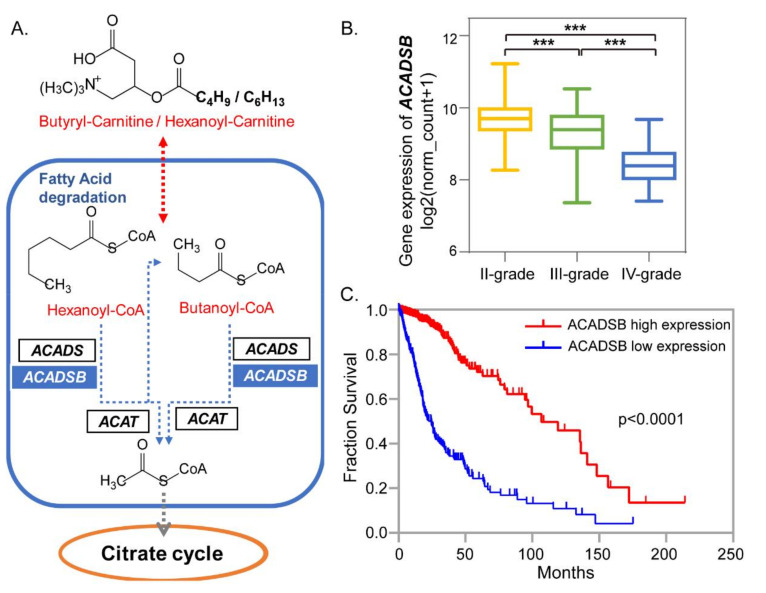
Short-chain acylcarnitine (C4-CN and C6-CN)-related changes for different grades of gliomas. (**A**) Degradation of short-chain acylcarnitines (in the form of acyl-CoAs) catalyzed by short/branched-chain acyl-coenzyme dehydrogenase (ACADSB) during fatty acid beta-oxidation. Red fonts represent up-regulation, and blue fonts represent down-regulation in high-grade gliomas. (**B**) The gene expression of ACADSB in different grades of glioma tissues according to TCGA database. (**C**) Kaplan–Meier analysis of patients having higher or lower ACADSB expression levels based on TCGA database. Significance level, *** *p* < 0.001.

For short-chain fatty acid β-oxidation, a series of acyl-coenzyme A (CoA) dehydrogenases played crucial roles. Elevated C4-CN and C6-CN were found in high-grade glioma tissues (Figure 3B). ACADS and short/branched-chain acyl-CoA dehydrogenase (ACADSB) are dehydrogenases that specifically target butanoyl-CoA (C4-CoA) and hexanoyl-CoA (C6-CoA) according to Kyoto Encyclopedia of Genes and Genomes (KEGG) (Figure S3), and strongly influence the contents of short-chain acylcarnitines (Figure 5A) [30]. The relationship between ACADSB (or ACADS) and glioma progression was further studied. The gene expressions of ACADS and ACADSB and clinical information were collected from 668 glioma patients in TCGA database. Mann–Whitney U tests were used to assess importance. ACADSB showed a significantly lower expression in high-grade gliomas (Figure 5B), whereas ACADS had no significant difference between grade II and grade III (Figure S4). Lower expression of ACADSB may lead to the accumulation of short-chain acylcarnitines, which further facilitate the growth and progression of gliomas. Moreover, Kaplan–Meier curves showed that patients with lower ACADSB had shorter overall survival time (Figure 5C). Similarly, the down-regulation of ACADSB was found to be correlated with worse overall survival of patients with kidney renal clear cell carcinoma or hepatocellular carcinoma [31,32]. This study indicates that lower ACADSB and more short-chain acylcarnitines are strongly related to glioma progression and further affect the glioma patients’ survival. It might be a key target for glioma treatment and prognosis evaluation.

## 3. Materials and Methods

### 3.1. Study Subjects

Seventy-six glioma patients were recruited for the current study with approval by the Scientific Research and Clinical Trial Ethics Committee of the First Affiliated Hospital of Zhengzhou University (ethic code: Scientific research-2016-LW-614). All subjects gave informed consent for inclusion prior to participation in the study.

Brain tumor tissues from all glioma patients and para-tumor tissues were collected during surgery. Samples of adjacent brain tissues were obtained as far from the glioma tissue as the operative field permitted. Pathological classifications were carried out by a neuropathologist according to the WHO grading. Tissue samples were immediately kept on ice within 1 h and then stored at −80 °C until analysis.

### 3.2. Metabolomics and Lipidomics Analysis

The GC-MS-based metabolomics, LC-MS-based metabolomics and LC-MS-based lipidomics analyses were performed on all tissue samples, and the detailed procedures have been described previously [33]. In brief, 10 mg of tissue was homogenized and extracted by methanol/methyl tert-butyl ether/water containing internal standards. After centrifugation, the solution was separated into the upper and lower layers. Then, 300 μL of the lower layer solution and 300 uL of the upper layer solution were drawn for GC-MS analysis and lipidomics analysis, respectively. Next, 150 μL of the lower layer solution and 200 μL of the upper layer solution were drawn and mixed for LC-MS-based metabolomics analysis. All samples were lyophilized and stored at −80 °C until analysis.

For GC-MS based metabolic profiling, the samples were derivatized with methoxyamine and followed by *N*-Methyl-*N*-(trimethylsilyl) trifluoroacetamide derivatization, then the derived mixture was separated by a DB-5 MS capillary column (30 m × 50 μm, 0.25 μm film thickness) (J&W Scientific, Folsom, CA, USA). Mass spectrometric analysis was performed on a QP2010 plus GC-MS system (Shimadzu, Kyoto, Japan).

For LC-MS based metabolic profiling, the samples were injected into a BEH C8 and a HSS T3 column (2.1 × 100 mm with 1.7 μm particle size) (Waters, Milford, MA, USA) coupled with triple TOF™ 5600 plus mass spectrometry (Applied Biosystems, Foster City, CA, USA) in positive and negative ionization modes, respectively.

For lipidomic analysis, the samples were injected into a BEH C8 column (2.1 × 100 mm with 1.7 μm particle size) (Waters, Milford, MA, USA) coupled with Q Exactive HF mass spectrometry (Thermo Fisher Scientific, Rockford, IL, USA) in positive and negative ionization modes.

All raw data were preprocessed using specialized software. For GC-MS metabolomics analysis, feature ions of metabolites were picked out and integrated by GC-MS solution software. For the LC-MS metabolomics and lipidomics analyses, automated peak detection, alignment and integration were performed using PeakView software 1.2.0.3 and TraceFinder 3.2, respectively. Next, peak areas were normalized to corresponding internal standards and tissue weight.

### 3.3. Gene Expression Analysis

TCGA lower grade glioma and glioblastoma (GBMLGG) cohorts were obtained from The Cancer Genome Atlas (TCGA) database, and mRNA expression and clinical information were downloaded from UCSC Xena [34]. In total, 249 grade II patients, 265 grade III patients, and 154 grade IV patients were involved in the current study. Statistical significance was performed using Mann–Whitney U test.

### 3.4. Statistics

PLS-DA analysis and permutation tests were performed using SIMCA-P 13.0. Mann–Whitney U test, Benjamini–Hochberg correction, and heatmap analysis of metabolites were performed using the Multi Experiment Viewer [35]. Correlation network analysis was performed based on the differential metabolites related to glioma grading by Cytospace 2.8.2. GraphPad Prism 6 was used for the box plot analysis and Kaplan–Meier curves analysis.

## 4. Conclusions

Collectively, we studied the metabolome alterations in different grades of gliomas based on the derived differential metabolites between gliomas and their para-tumor tissues. The paired samples in the scheme reduced the interference from individual differences on metabolic characteristics and made the discovery more credible. Short-chain acylcarnitines and LPE metabolism showed significant alterations as the tumor progressed. Combined with gene expression data from TCGA database, lower ACADSB responsible for the elevated short-chain acylcarnitines was found to be strongly related to glioma progression. However, further studies are needed to elucidate the detailed molecular interactions between ACASB and glioma progression.

## Figures and Tables

**Table 1:** Clinical characteristics of the glioma patients for glioma grading study.

Gliomas	WHO Grading		
	II	III	IV
Sample No.	30	21	18
Gender, male/female	14/16	9/12	4/14
Age, medium (min~max)	43.8 (24~70)	47 (20~76)	55.7 (36~71)

## Supplementary Materials

The following are available online at https://www.mdpi.com/2218-1989/10/12/478/s1, Figure S1: The compound classifications of 668 metabolites identified in gliomas and para-tumor tissues, Figure S2: The context of acylcarnitines in different grades of glioma tissues, Figure S3: Fatty acid degradation pathway in KEGG, Figure S4: The gene expression of ACADS in different grades of glioma tissues, Table S1: The chromatogram-mass spectrometry information of 668 identified metabolites.
