# Supplementary material for: Metabolic Alterations Related to Glioma Grading Based on Metabolomics and Lipidomics Analyses
Source: Metabolites. 2020 Nov 24;10(12):478. doi: 10.3390/metabo10120478 (PMC7760389; doi:10.3390/metabo10120478)
Supplement: Supplementary file 1 [file metabolites-10-00478-s001.pdf]

Metabolic Alterations related to Glioma Grading Based on Metabolomics and Lipidomics analyses

Di Yu [1,3], Qiuhui Xuan [1,3], Chaoqi Zhang [2], Chunxiu Hu [1], Yanli Li [1], Xinjie Zhao [1], Shasha Liu [2], Feifei Ren [2], Yi Zhang [2,*], Lina Zhou [1,*] and Guowang Xu [1,*]

[1] CAS Key Laboratory of Separation Science for Analytical Chemistry, Dalian Institute of Chemical Physics, Chinese Academy of Sciences, Dalian 116023, China; yudi_1808@dicp.ac.cn (D.Y.); xuanqiuhui@dicp.ac.cn (Q.X.); hucx@dicp.ac.cn (C.H.); liyanli@dicp.ac.cn (Y.L.); xj_zhao1@126.com (X.Z.)

[2] Biotherapy Center and Cancer Center, The First Affiliated Hospital of Zhengzhou University, Zhengzhou 450052, China; chaoqizhang1917@163.com (C.Z.); liushasha910729@163.com (S.L.); feifeiren312@163.com (F.R.)

[3] University of Chinese Academy of Sciences, Beijing 100049, China;

* Correspondence: yizhang@zzu.edu.cn (Y.Z.); zhouln@dicp.ac.cn (L.Z.); xugw@dicp.ac.cn (G.X.); Tel.: +86-411-84379530

Contents

Figure S1. 668 metabolites identified in gliomas and para-tumor tissues … … … … … … …s-2

Figure S2. The context of acylcarnitines in different grades of glioma tissues … … … … … s-3

Figure S3. Fatty acid degradation pathway in KEGG … … … … … … … … … … … … … … …s-4

Figure S4. The gene expression of ACADS in different grades of glioma tissues … … … … s-5

Table S1. The chromatogram-mass spectrometry information of 668 identified metabolites by integrated metabolomics and lipidomics analyses … … …… … …… … …… … …… … …... s-6.

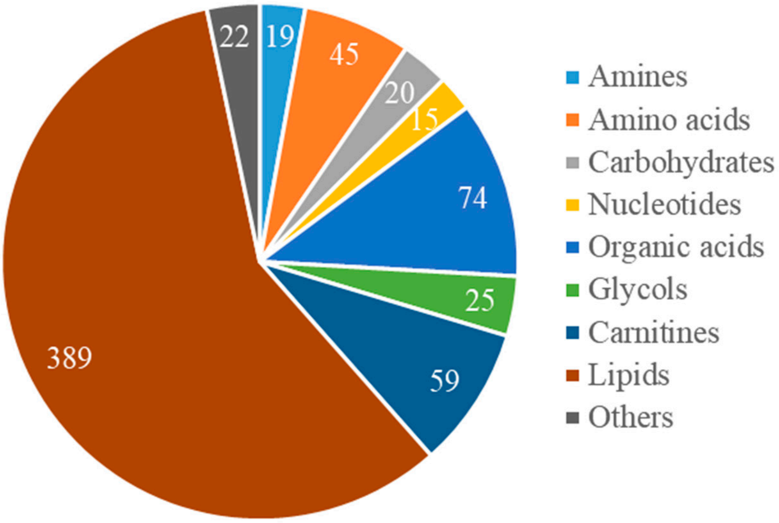

Figure S1. 668 metabolites identified in gliomas and para-tumor tissues.

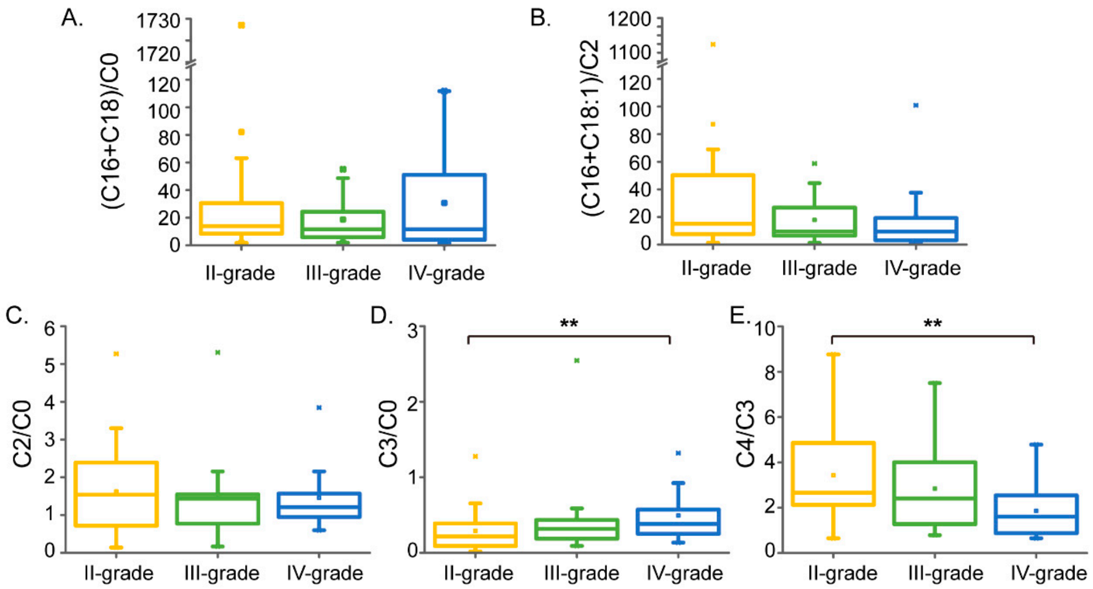

Figure S2. The context of acylcarnitines in different grades of glioma tissues. Significance level, **p<0.01

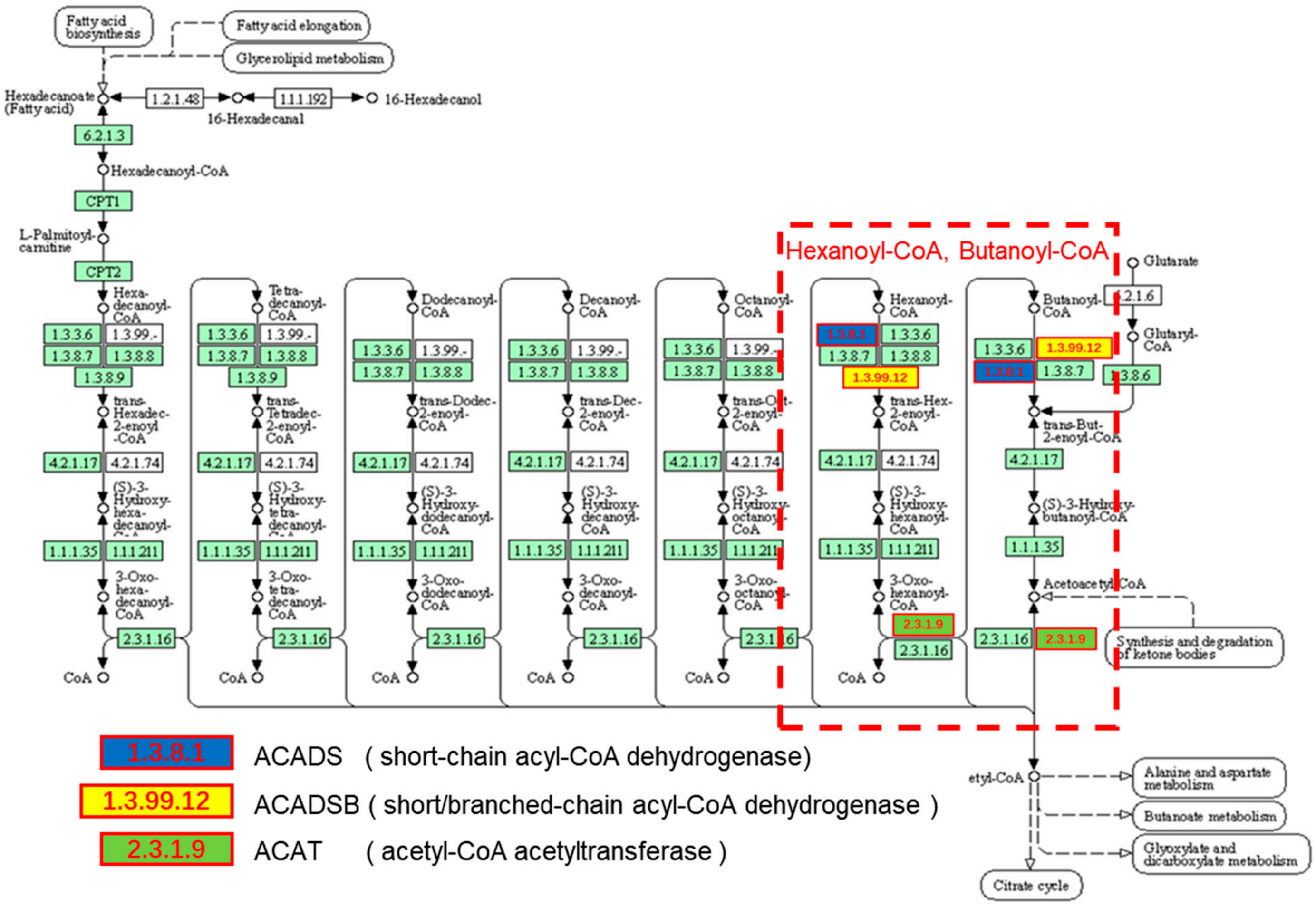

Figure S3. Fatty acid degradation pathway in KEGG. The specific enzymes for beta-oxidation of short-chain acyl-CoA were colored.

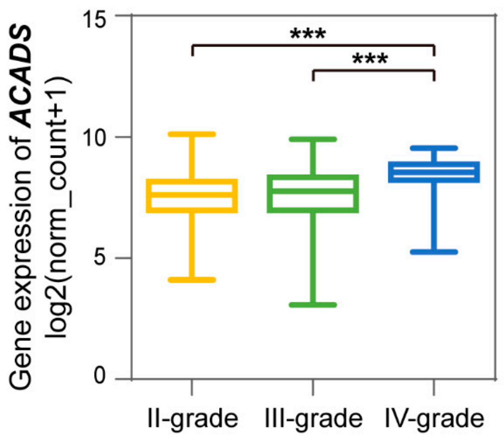

Figure S4. The gene expression of ACADS in different grades of glioma tissues according to TCGA database. Significance level, ***p<0.001.

Table S1. The chromatogram-mass spectrometry information of 668 identified metabolites by integrated metabolomics and lipidomics analyses. Elevated or decreased trends of the differential metabolites between gliomas and para-tumor tissues were labeled with their significance level. p<0.05 and FDR<0.05; ** P<0.01 and FDR<0.05; *** p<0.001 and FDR<0.05.

ID	Platform	Compounds[adduct ion]	m/z	t_R(min)	Gliomas
1	GC-MS	Pyroglutamic acid	156	20.41	
2	LC-MS-neg	L-Glutathione[-H]	611.142	0.62	
3	LC-MS-neg	Glutamine[-H]	145.062	0.69	
4	LC-MS-neg	Glutamic acid[-H]	146.047	0.63	
5	LC-MS-neg	3-Hydroxymethylglutaric acid[-H]	161.046	0.70	
6	LC-MS-neg	N-Acetylglutamic acid[-H]	188.055	0.62	
7	LC-MS-pos	L-Cysteine-Glutathione Disulfide[+H]	427.095	0.73	
8	LC-MS-pos	N-metyl-L-glutamic acid[+H]	162.076	0.80	Elevated *
9	LC-MS-pos	Glu-Leu[+H]	261.147	3.04	
10	GC-MS	Isoleucine	158	14.51	
11	LC-MS-pos	Leucine[+H]	132.102	1.20	
12	LC-MS-pos	Ala-Leu[+H]	203.138	0.78	
13	LC-MS-pos	Pro-leu[+H]	229.155	0.86	
14	GC-MS	N-Acetyl-L-Lysine	174	27.88	
15	LC-MS-neg	N-Acetylaspartic acid[-H]	174.041	0.61	
16	LC-MS-neg	N-Acetylmethionine[-H]	190.054	2.78	
17	LC-MS-neg	Na-Acetylarginine[-H]	215.034	0.68	
18	LC-MS-pos	O-acetylSerine[+H]	148.06	0.77	
19	LC-MS-pos	N8-Acetylspermidine[+H]	188.175	0.64	
20	GC-MS	Tyrosine	179	28.56	
21	GC-MS	Alanine	116	9.13	
22	GC-MS	Proline	142	14.59	
23	GC-MS	Glycine	174	14.83	
24	GC-MS	Serine	204	16.37	
25	GC-MS	Threonine	218	17.03	
26	GC-MS	Beta-Alanine	248	18.11	
27	GC-MS	Aspartic acid	232	20.48	
28	GC-MS	Trans-4-Hydroxyproline	230	20.58	
29	GC-MS	Cysteine	220	21.29	
30	GC-MS	Asparagine	116	23.96	
31	LC-MS-neg	Lactamide[-H]	88.0436	0.76	
32	LC-MS-neg	S-Adenosy-L-Homocysteine[-H]	383.114	3.48	
33	LC-MS-neg	Ornithine[-H]	131.083	0.76	
34	LC-MS-neg	Ala-Gly[-H]	145.063	0.69	
35	LC-MS-neg	3-(1-Pyrazolyl)-alanine[-H]	154.064	0.77	
36	LC-MS-neg	Theanine[-H]	173.105	0.76	
37	LC-MS-neg	N,N-Dimethylglycine[-H]	102.058	0.63	
38	LC-MS-neg	O-succiny-L-homoserine[-H]	218.066	0.62	
39	LC-MS-neg	Homoserine[-H]	118.052	0.70	
40	LC-MS-pos	S-Adenosyl-L-methionine[+H]	399.145	0.64	
41	LC-MS-pos	Methionine[+H]	150.058	1.00	
42	LC-MS-pos	Lysine[+H]	147.113	0.64	
43	LC-MS-pos	Histidine[+H]	156.077	0.64	
44	LC-MS-pos	Phenylalanine[+H]	166.086	1.60	
45	LC-MS-pos	Arginine[+H]	175.119	0.66	
46	LC-MS-pos	Citrulline[+H]	176.104	0.74	
47	LC-MS-pos	Tryptophan[+H]	205.097	2.46	
48	LC-MS-pos	L-Homoserine lactone[+H]	102.055	0.74	
49	LC-MS-pos	Gly-Tyr[+H]	239.107	0.72	
50	LC-MS-pos	glycated valine[+H]	296.067	0.71	
51	LC-MS-pos	Valine[+H]	118.087	0.76	
52	GC-MS	Ethanolamine	174	13.74	
53	GC-MS	Meso-Erythritol	217	20.16	
54	GC-MS	O-Phosphoethanolamine	299	26.40	
55	LC-MS-pos	3,4-dihydroxy-DL-phenylalanine[+H	198.087	0.74	
56	LC-MS-pos	Nicotinamide[+H]	123.056	0.99	
57	GC-MS	Putrescine	174	25.37	
58	LC-MS-pos	Pyridoxamine[+H]	169.093	0.66	
59	LC-MS-pos	Spermidine[+H]	146.165	0.60	
60	LC-MS-pos	Spermine[+H]	203.223	0.64	
61	GC-MS	Urea	189	13.22	
62	LC-MS-pos	Creatine[+H]	132.076	0.74	
63	LC-MS-pos	creatinine[+H]	114.065	0.74	
64	GC-MS	2-Aminobutyric acid	130	11.11	
65	GC-MS	4-Aminobutanoic acid	304	20.66	
66	GC-MS	3-Aminoisobutyric acid	174	18.83	
67	GC-MS	2,3,4-Trihydroxybutyric acid	292	21.44	
68	LC-MS-neg	13-cis-acitretin[-H]	325.184	15.92	
69	LC-MS-neg	2-Aminoethylphosphonic acid[-H]	124.009	0.68	
70	LC-MS-pos	3-Aminosalicylic acid[+H]	154.057	0.74	
71	GC-MS	3-Hydroxybutanoic acid	147	10.80	
72	GC-MS	3-Phosphoglyceric acid	299	26.97	
73	GC-MS	Aminomalonic acid	218	19.19	
74	LC-MS-pos	Argininosuccinic acid[+H]	291.127	0.74	
75	GC-MS	Citric acid	273	27.23	
76	LC-MS-neg	Dimethyluric acid[-H]	195.053	0.64	
77	LC-MS-neg	DL-2-Aminoadipic acid[-H]	160.063	0.63	
78	LC-MS-neg	malic acid[-H]	133.015	0.60	
79	LC-MS-neg	Fumaric acid[-H]	115.005	0.60	
80	LC-MS-pos	Glucoheptonic acid[+H]	249.053	1.07	
81	GC-MS	Hypotaurine	188	22.26	
82	LC-MS-neg	Isocitric acid[-H]	191.02	0.55	
83	LC-MS-neg	Lactic acid[-H]	89.0268	0.69	
84	GC-MS	Phosphoenol pyruvic acid	369	22.33	
85	LC-MS-neg	Orthophosphoric acid[-H]	96.9712	0.60	
86	GC-MS	Oxalic acid	147	10.05	
87	LC-MS-pos	pantothenic acid[+H]	220.119	1.92	
88	GC-MS	Pyruvic acid	174	7.67	
89	GC-MS	Succinic acid	147	15.16	
90	LC-MS-neg	Uric acid[-H]	167.023	0.76	
91	LC-MS-pos	α-Keto-γ-(methylthio)butyric acid[+I	149.023	15.16	
92	GC-MS	Dehydroascorbic acid	173	27.72	
93	GC-MS	1,4-Benzenedicarboxylic acid	295	26.58	
94	GC-MS	2-Aminoadipic acid	188	23.08	
95	LC-MS-neg	2-Hydroxyglutaric acid[-H20-H]	129.02	0.59	
96	GC-MS	2-ketoglutaric acid	198	21.86	
97	GC-MS	Azelaic acid	317	26.82	
98	GC-MS	1,3-Propanediol	115	7.82	
99	LC-MS-pos	Calcifediol[+H]	401.345	18.94	
100	LC-MS-pos	1-Hexadecanol[+H]	265.253	18.74	
101	LC-MS-pos	7-Ketocholesterol[+H]	401.34	18.94	
102	LC-MS-neg	allo-inositol[-H]	201.039	0.68	
103	GC-MS	Cholesterol	368	48.58	
104	LC-MS-pos	Dihydrosphingosine[+H]	302.304	13.30	
105	LC-MS-neg	Dulcitol[-H]	181.073	0.72	
106	GC-MS	Ethylene glycol	147	5.95	
107	LC-MS-neg	Etiocholanolone sulfate[-H]	369.174	12.38	
108	GC-MS	Mannitol	319	29.40	
109	GC-MS	Hexyl alcohol	159	6.08	
110	GC-MS	Myo-Inositol	305	32.47	
111	GC-MS	Myo-inositol phosphate	318	38.08	
112	LC-MS-pos	Palmitoylethanolamide[+H]	300.29	12.98	
113	LC-MS-pos	Phytosphingosine[+H]	318.301	10.95	Decreased**
114	GC-MS	scyllo-Inositol	318	31.32	
115	GC-MS	Sorbitol	319	29.53	
116	GC-MS	Glyceraldehyde	160	12.54	
117	GC-MS	Glycerol	205	14.04	
118	GC-MS	Glyceraldehyde 3-phosphate	299	25.12	
119	GC-MS	Glyceric acid	292	15.56	
120	GC-MS	Glycerol-2-phosphate	243	25.21	
121	GC-MS	Glycerol-3-phosphate	357	26.02	
122	LC-MS-neg	Glycerophosphate ethanolamine[-H]	214.047	0.67	
123	LC-MS-pos	Diisooctyl phthalate[+H]	391.284	20.73	
124	LC-MS-pos	Methyl arachidonate[+H]	319.264	19.74	
125	LC-MS-pos	Monoethylhexyl phthalate[+H]	279.159	15.12	
126	GC-MS	Dihydroxyacetone phosphate	315	25.87	
127	LC-MS-pos	Farnesyl acetone[+H]	263.239	17.78	
128	LC-MS-neg	Cystathionine[-H]	221.059	0.65	
129	LC-MS-pos	Proline betaine[+H]	144.102	0.78	
130	LC-MS-pos	Choline[+H]	104.107	0.72	
131	LC-MS-pos	Choline glycerophosphate[+H]	258.111	0.74	
132	GC-MS	Mimosine	188	11.87	
133	LC-MS-pos	Octopine[+H]	247.145	3.06	
134	LC-MS-pos	Alpha-Lactose[+H]	365.107	0.74	

(continued)

ID	Platform	Compounds[adduct ion]	m/z	t_R(min)	Gliomas	ID	Platform	Compounds[adduct ion]	m/z	t_R(min)	Gliomas
135	LC-MS-pos	Altrose[+H]	203.052	0.74		202	LC-MS-pos	Carnitine C14:1-3[+H]	370.295	12.80	
136	GC-MS	D-erythrose 4-phosphate	357	33.09		203	LC-MS-pos	Carnitine C14:2[+H]	368.28	11.66	
137	GC-MS	D-Fructose 6-phosphate	315	36.36		204	LC-MS-pos	Carnitine C15:0[+H]	386.327	13.80	
138	GC-MS	D-Glucopyranose	204	30.47		205	LC-MS-pos	Carnitine C16:0[+H]	400.342	14.44	
139	GC-MS	D-Glucose-6-phosphate	387	36.56		206	LC-MS-pos	Carnitine C16:1-1[+H]	398.327	13.66	
140	GC-MS	D-ribose-5-phosphate	299	32.87		207	LC-MS-pos	Carnitine C16:1-2[+H]	398.327	14.14	
141	GC-MS	Maltose	361	43.05		208	LC-MS-pos	Carnitine C16:2[+H]	396.311	12.92	
142	GC-MS	Maltotriose	361	53.77		209	LC-MS-pos	Carnitine C16:3[+H]	394.295	12.50	
143	GC-MS	Mannose	204	28.89		210	LC-MS-pos	Carnitine C17:0[+H]	414.358	15.07	
144	GC-MS	Fructose	103	28.27		211	LC-MS-pos	Carnitine C17:1[+H]	412.342	14.26	
145	GC-MS	Galactonic acid	333	30.76		212	LC-MS-pos	Carnitine C18:0[+H]	428.373	15.66	
146	LC-MS-neg	Glucosamine 6-phosphate[-H]	258.04	0.63		213	LC-MS-pos	Carnitine C18:1[+H]	426.358	14.86	
147	GC-MS	Glucose	319	28.77		214	LC-MS-pos	Carnitine C18:2[+H]	424.342	14.10	
148	GC-MS	Isopropyl-1-thiogalactopyranoside	361	30.00		215	LC-MS-pos	Carnitine C18:3[+H]	422.327	13.56	
149	LC-MS-neg	N-Acetylglucosamine-6-Phosphate[	300.049	0.61		216	LC-MS-pos	Carnitine C19:0[+H]	442.389	16.23	
150	GC-MS	Ribose	307	24.15		217	LC-MS-pos	Carnitine C19:1[+H]	440.373	15.41	
151	GC-MS	Sucrose	361	41.60		218	LC-MS-pos	Carnitine C20:0[+H]	456.405	16.77	
152	LC-MS-pos	UDP-N-acetyl-glucosamine[+H]	608.09	0.90		219	LC-MS-pos	Carnitine C20:1[+H]	454.389	15.96	
153	LC-MS-neg	Uridine diphosphate glucose[-H]	565.045	0.63		220	LC-MS-pos	Carnitine C20:2[+H]	452.373	15.24	
154	GC-MS	2,6-Dihydroxy-9H-Purine	353	31.19		221	LC-MS-pos	Carnitine C20:3[+H]	450.358	14.62	
155	LC-MS-pos	2-Aminoadenosine[+H]	305.097	1.02	Decreased**	222	LC-MS-pos	Carnitine C22:0[+H]	484.436	17.77	
156	LC-MS-pos	8-Hydroxy-2'-deoxyguanosine[+H]	284.099	1.00		223	LC-MS-pos	Carnitine C22:2[+H]	480.405	16.29	
157	GC-MS	Adenine	264	28.13		224	LC-MS-pos	Carnitine C22:3[+H]	478.389	15.66	
158	LC-MS-pos	Guanine[+H]	152.056	1.00		225	LC-MS-pos	Carnitine C22:6[+H]	472.342	14.19	
159	LC-MS-pos	Hypoxanthine[+H]	137.046	0.98		226	LC-MS-pos	Carnitine C24:0[+H]	512.467	18.68	
160	LC-MS-neg	Hypoxanthine-9-arabinofuranosine[-	267.073	2.63		227	LC-MS-pos	Carnitine C24:1[+H]	510.452	17.92	
161	LC-MS-neg	Oxypurinol[-H]	151.028	1.06		228	LC-MS-pos	Carnitine C26:0[+H]	540.499	19.47	
162	LC-MS-pos	Xanthine[+H]	153.041	1.00		229	LC-MS-pos	Carnitine C26:1[+H]	538.483	18.78	
163	LC-MS-pos	Adenosine[+H]	268.104	1.00	Elevated **	230	LC-MS-pos	Carnitine ph-C14[+H]	448.342	14.20	
164	LC-MS-pos	adenosine 5'-monophosphate[+H]	348.07	0.75		231	LC-MS-pos	Carnitine ph-C16[+H]	476.373	15.14	
165	LC-MS-neg	Gamma-Glu-Cys[-H]	275.088	0.59		232	LC-MS-pos	Carnitine isoC5-OH[+H]	262.165	1.22	
166	GC-MS	Inosine	230	40.67		233	LC-MS-pos	Carnitine C6-OH[+H]	276.181	2.90	
167	LC-MS-pos	N6-Succinyl adenosine[+H]	384.117	1.54		234	LC-MS-pos	Carnitine C8-OH[+H]	304.212	5.44	
168	GC-MS	Uridine	217	38.62		235	LC-MS-pos	Carnitine C12-OH[+H]	360.275	9.91	
169	GC-MS	2-Hydroxypyridine	152	7.26		236	LC-MS-pos	Carnitine C14-OH[+H]	388.306	11.56	
170	GC-MS	3-Hydroxypyridine	152	10.07		237	LC-MS-pos	Carnitine C16-OH[+H]	416.337	12.98	
171	GC-MS	Uracil	241	15.72		238	LC-MS-pos	Carnitine C16:2-OH[+H]	412.306	11.54	
172	LC-MS-pos	Indoline[+H]	120.081	1.60		239	LC-MS-pos	Carnitine C18-OH[+H]	444.368	14.31	
173	LC-MS-pos	pyrrolidine[+H]	72.0824	0.82		240	LC-MS-pos	Carnitine C18:1-OH[+H]	442.353	13.54	
174	LC-MS-neg	p-Cresol sulfate[-H]	187.007	5.40		241	LC-MS-pos	Carnitine C18:2-OH[+H]	440.337	12.77	
175	LC-MS-pos	PhenolRed[+H]	355.065	6.13		242	LC-MS-pos	Carnitine C18:3-OH[+H]	438.321	12.36	
176	LC-MS-pos	3-Methylisoquinoline[+H]	144.08	2.46		243	LC-MS-pos	Carnitine C20-OH[+H]	472.4	15.54	
177	LC-MS-pos	4-Hydroxyquinoline[+H]	146.06	2.46		244	LC-MS-pos	Carnitine C20:1-OH[+H]	470.384	14.72	
178	Lipid-pos	Co(Q10)[+H]	863.693	11.88		245	LC-MS-pos	Carnitine C20:2-OH[+H]	468.368	14.04	
179	Lipid-pos	ChE(18:2)[+NH4]	666.619	12.66		246	LC-MS-pos	Carnitine C20:3-OH[+H]	466.353	13.44	
180	LC-MS-neg	5-cholanic acid[-H]	359.296	17.56		247	LC-MS-pos	Carnitine C5:DC[+H]	276.144	1.06	
181	LC-MS-neg	Dehydroepiandrosterone Sulfate[-H]	367.158	11.32		248	Lipid-neg	FFA 13:0[-H]	213.186	1.52	Decreased***
182	LC-MS-neg	Deoxycholate[-H]	391.284	14.55		249	Lipid-neg	FFA 14:0[-H]	227.202	1.68	
183	LC-MS-neg	Glycochenodeoxycholate[-H]	448.306	14.19		250	Lipid-neg	FFA 14:1[-H]	225.186	1.50	
184	LC-MS-neg	Glycodeoxycholate[-H]	448.305	14.40		251	Lipid-neg	FFA 15:0[-H]	241.217	1.88	
185	LC-MS-neg	Ursodeoxycholic acid[-H]	391.286	14.78		252	Lipid-neg	FFA 16:0[-H]	255.233	2.12	
186	GC-MS	1-Monopalmitin	371	40.99		253	Lipid-neg	FFA 16:1[-H]	253.217	1.80	
187	LC-MS-pos	Glycerol-1-Hexadecanoate[+H]	331.287	17.34		254	Lipid-neg	FFA 17:0[-H]	269.249	2.42	Decreased*
188	GC-MS	Monostearin	399	43.83	Decreased*	255	Lipid-neg	FFA 17:1[-H]	267.233	2.00	Decreased***
189	LC-MS-pos	Carnitine [+H]	162.113	0.73		256	Lipid-neg	FFA 18:0[-H]	283.264	2.81	
190	LC-MS-pos	Carnitine C2:0[+H]	204.123	0.80	Elevated **	257	Lipid-neg	FFA 18:1[-H]	281.249	2.27	
191	LC-MS-pos	Carnitine C3:0[+H]	218.139	1.18	Elevated *	258	Lipid-neg	FFA 18:2[-H]	279.233	1.88	
192	LC-MS-pos	Carnitine C4:0[+H]	232.154	2.18	Elevated **	259	Lipid-neg	FFA 18:3[-H]	277.217	1.68	
193	LC-MS-pos	Carnitine C5:0-1[+H]	246.17	3.51		260	Lipid-neg	FFA 19:0[-H]	297.28	3.26	Decreased**
194	LC-MS-pos	Carnitine C5:0-2[+H]	246.17	3.63		261	Lipid-neg	FFA 20:0[-H]	311.296	3.77	
195	LC-MS-pos	Carnitine C5:1[+H]	244.154	3.11		262	Lipid-neg	FFA 20:1[-H]	309.28	2.97	Decreased**
196	LC-MS-pos	Carnitine C6:0[+H]	260.186	5.24	Elevated ***	263	Lipid-neg	FFA 20:2[-H]	307.264	2.47	
197	LC-MS-pos	Carnitine C12:0[+H]	344.28	11.66		264	Lipid-neg	FFA 20:3[-H]	305.249	2.15	
198	LC-MS-pos	Carnitine C12:1[+H]	342.264	10.88		265	Lipid-neg	FFA 20:4[-H]	303.233	1.82	
199	LC-MS-pos	Carnitine C14:0[+H]	372.311	13.12		266	Lipid-neg	FFA 20:5[-H]	301.217	1.58	
200	LC-MS-pos	Carnitine C14:1-1[+H]	370.295	12.28		267	Lipid-neg	FFA 21:0[-H]	325.311	4.33	
201	LC-MS-pos	Carnitine C14:1-2[+H]	370.295	12.46		268	Lipid-neg	FFA 22:0[-H]	339.327	4.90	

(continued)

ID	Platform	Name	m/z	t_R(min)	Gliomas	ID	Platform	Name	m/z	t_R(min)	Gliomas
**269**	Lipid-neg	FFA 22:1[-H]	337.311	3.89		**335**	Lipid-pos	LPC 18:2[+H]	520.341	1.58	
**270**	Lipid-neg	FFA 22:2[-H]	335.296	3.19		**336**	LC-MS-pos	LPC 20:1[+H]	550.388	16.28	Decreased***
**271**	Lipid-neg	FFA 22:3[-H]	333.28	2.72		**337**	LC-MS-pos	LPC 20:2[+H]	548.371	15.42	Decreased*
**272**	Lipid-neg	FFA 22:4[-H]	331.264	2.23		**338**	Lipid-pos	LPC 20:3[+H]	546.355	2.32	Decreased**
**273**	Lipid-neg	FFA 22:5[-H]	329.248	1.95		**339**	LC-MS-pos	LPC 20:3 sn-1[+H]	546.355	14.22	
**274**	Lipid-neg	FFA 22:6[-H]	327.233	1.68		**340**	LC-MS-pos	LPC 20:3 sn-2[+H]	546.355	14.58	
**275**	Lipid-neg	FFA 23:0[-H]	353.343	5.53		**341**	LC-MS-pos	LPC 20:4[+H]	544.34	14.00	
**276**	Lipid-neg	FFA 24:0[-H]	367.358	6.09		**342**	LC-MS-pos	LPC 22:4[+H]	572.371	15.40	
**277**	Lipid-neg	FFA 24:1[-H]	365.343	4.96		**343**	LC-MS-pos	LPC 22:6[+H]	568.339	14.25	
**278**	Lipid-neg	FFA 25:0[-H]	381.374	6.65		**344**	Lipid-pos	LPC 24:1[+H]	606.451	4.18	Decreased**
**279**	Lipid-neg	FFA 26:0[-H]	395.389	7.20		**345**	Lipid-pos	LPC 26:1[+H]	634.482	5.24	Decreased**
**280**	LC-MS-pos	FFAD C14:0[+H]	228.233	15.09	Decreased*	**346**	Lipid-pos	LPC 26:0[+H]	636.497	6.42	Decreased**
**281**	LC-MS-pos	FFAD C18:0[+H]	284.296	18.35		**347**	LC-MS-pos	LPE 16:0 sn-1[+H]	454.293	14.10	Decreased***
**282**	LC-MS-pos	FFAD C20:1[+H]	310.312	18.74	Decreased*	**348**	LC-MS-pos	LPE 16:0 sn-2[+H]	454.293	14.39	Decreased***
**283**	Lipid-pos	Cer(d16:1/16:0)[+H]	510.489	7.03		**349**	LC-MS-pos	LPE 16:1[+H]	452.277	13.24	Decreased***
**284**	Lipid-pos	Cer(d18:0/16:0)[+H]	540.536	8.19	Elevated ***	**350**	LC-MS-pos	LPE 18:1 sn-1[+H]	480.309	14.64	Decreased***
**285**	Lipid-pos	Cer(d18:0/24:2)[+H]	648.63	10.42		**351**	LC-MS-pos	LPE 18:1 sn-2[+H]	480.309	14.92	Decreased***
**286**	Lipid-neg	Cer(d18:1/14:0)[+H]	508.471	6.96		**352**	LC-MS-pos	LPE 18:2 sn-1[+H]	478.293	13.82	Decreased**
**287**	Lipid-pos	Cer(d18:1/16:0)[+H]	538.52	7.95		**353**	LC-MS-pos	LPE 18:2 sn-2[+H]	478.293	14.06	Decreased**
**288**	Lipid-neg	Cer(d18:1/17:0)[+H]	550.519	8.30	Decreased***	**354**	LC-MS-pos	LPE 20:1 sn-1[+H]	508.342	15.96	Decreased***
**289**	Lipid-pos	Cer(d18:1/18:0)[+H]	566.551	8.72		**355**	LC-MS-pos	LPE 20:1 sn-2[+H]	508.342	16.23	Decreased***
**290**	Lipid-pos	Cer(d18:1/19:0)[+H]	580.567	9.17	Decreased***	**356**	LC-MS-pos	LPE 20:2[+H]	506.327	15.24	Decreased***
**291**	Lipid-pos	Cer(d18:1/22:0)[+H]	622.614	10.26		**357**	LC-MS-neg	LPE 20:3[-H]	502.291	17.19	Decreased***
**292**	Lipid-pos	Cer(d18:1/22:1)[+H]	620.599	9.60	Decreased**	**358**	LC-MS-pos	LPE 20:4 sn-1[+H]	502.293	13.93	Decreased***
**293**	Lipid-pos	Cer(d18:1/22:3)[+H]	616.565	9.53		**359**	LC-MS-pos	LPE 20:4 sn-2[+H]	502.293	14.18	Decreased*
**294**	Lipid-pos	Cer(d18:1/24:0)[+H]	650.646	10.89		**360**	LC-MS-pos	LPE 22:1[+H]	536.374	17.24	Decreased***
**295**	Lipid-pos	Cer(d18:1/24:1)[+H]	648.63	10.20	Decreased***	**361**	LC-MS-pos	LPE 22:2[+H]	534.356	16.45	Decreased***
**296**	Lipid-pos	Cer(d18:1/24:2)[+H]	646.615	9.62		**362**	LC-MS-pos	LPE 22:3 sn-1[+H]	532.34	15.62	Decreased***
**297**	Lipid-pos	Cer(d18:1/26:1)[+H]	676.662	10.84	Decreased***	**363**	LC-MS-pos	LPE 22:3 sn-2[+H]	532.34	15.88	Decreased**
**298**	Lipid-pos	Cer(d18:2/16:0)[+H]	536.504	7.19		**364**	LC-MS-pos	LPE 22:5[+H]	528.309	14.35	Decreased***
**299**	Lipid-pos	Cer(d18:2/18:0)[+H]	564.536	8.09		**365**	Lipid-pos	LPS(38:6)[+H]	794.534	6.03	
**300**	Lipid-pos	Cer(d18:2/20:0)[+H]	592.567	8.93		**366**	Lipid-pos	PC 28:0[+H]	678.508	5.94	
**301**	Lipid-pos	Cer(d18:2/22:0)[+H]	620.598	9.78		**367**	Lipid-pos	PC 29:0[+H]	692.523	6.38	Decreased**
**302**	Lipid-neg	Cer(d18:2/24:0)[+H]	646.611	10.43		**368**	Lipid-pos	PC 30:0[+H]	706.539	6.85	Decreased**
**303**	Lipid-pos	Cer(d18:2/26:0)[+H]	676.662	10.65		**369**	Lipid-pos	PC 30:1[+H]	704.523	6.33	
**304**	Lipid-pos	Cer(d20:1/18:0)[+H]	594.583	9.50		**370**	Lipid-pos	PC 31:0[+H]	720.555	7.27	Decreased**
**305**	Lipid-pos	CerG1(d18:0/18:0)[+H]	730.62	8.43		**371**	Lipid-pos	PC 31:2[+H]	716.523	7.39	Decreased**
**306**	Lipid-pos	CerG1(d18:1/16:0)[+H]	700.573	7.27		**372**	Lipid-pos	PC 31:3[+H]	714.507	6.73	Decreased**
**307**	Lipid-pos	CerG1(d18:1/18:0)[+H]	728.604	8.13	Decreased**	**373**	Lipid-pos	PC 32:0[+H]	734.57	6.13	Decreased***
**308**	Lipid-pos	CerG1(d18:1/24:0)[+H]	812.699	10.40	Decreased*	**374**	Lipid-pos	PC 32:1[+H]	732.554	7.01	Decreased**
**309**	Lipid-pos	CerG1(d18:1/24:1)[+H]	810.683	9.66	Decreased***	**375**	Lipid-pos	PC 32:3[+H]	728.523	6.08	
**310**	Lipid-pos	CerG1(d18:1/26:1)[+H]	838.715	10.36	Decreased**	**376**	Lipid-pos	PC 33:0[+H]	748.586	8.06	Decreased***
**311**	Lipid-pos	CerG2(d18:1/16:0)[+H]	862.625	6.95		**377**	Lipid-pos	PC 33:1[+H]	746.571	7.55	Decreased***
**312**	Lipid-pos	CerG2(d18:1/24:1)[+H]	972.736	9.50	Decreased***	**378**	Lipid-pos	PC 33:2[+H]	744.555	6.77	Decreased***
**313**	Lipid-neg	CL(18:1/18:0/18:0/20:0)[-H]	1490.11	8.88		**379**	Lipid-pos	PC(15:1_18:1)[+H]	744.555	8.26	Decreased**
**314**	Lipid-neg	CL(18:2/18:2/18:2/18:2)[-H]	1447.96	11.16		**380**	Lipid-pos	PC 33:4[+H]	740.523	7.34	Decreased*
**315**	Lipid-neg	CL(20:4/18:0/18:1/20:0)[-H]	1510.08	8.21		**381**	Lipid-pos	PC 34:0[+H]	762.602	8.58	Decreased**
**316**	Lipid-neg	CL(21:0/16:0/16:0/22:6)[-H]	1494.05	7.05		**382**	Lipid-pos	PC 34:1[+H]	760.586	7.87	Decreased***
**317**	Lipid-neg	CL(22:2/18:0/18:1/20:4)[-H]	1534.08	8.21		**383**	Lipid-pos	PC 34:2[+H]	758.57	7.27	Decreased**
**318**	Lipid-neg	CL(23:0/18:0/18:0/22:4)[-H]	1582.17	8.73		**384**	Lipid-pos	PC(16:0_18:3)[+H]	756.552	7.69	Decreased***
**319**	Lipid-neg	CL(23:1/16:0/18:0/18:1)[-H]	1502.11	8.09		**385**	Lipid-pos	PC 34:4[+H]	754.54	6.25	
**320**	Lipid-neg	CL(23:1/18:1/18:1/18:1)[-H]	1526.11	8.07		**386**	Lipid-pos	PC(18:4_16:0)[+H]	754.537	7.15	Decreased**
**321**	LC-MS-pos	LPC 14:0 sn-1[+H]	468.309	12.67	Decreased**	**387**	Lipid-pos	PC 35:1[+H]	774.602	8.36	Decreased***
**322**	LC-MS-pos	LPC 14:0 sn-2[+H]	468.309	12.97	Decreased**	**388**	Lipid-pos	PC 35:2[+H]	772.586	7.67	Decreased***
**323**	Lipid-pos	LPC 15:1[+H]	480.309	2.01	Decreased***	**389**	Lipid-pos	PC 35:3[+H]	770.572	8.50	
**324**	LC-MS-pos	LPC 16:0 sn-1[+H]	496.339	14.16	Decreased***	**390**	Lipid-pos	PC 35:4[+H]	768.555	6.75	Decreased**
**325**	LC-MS-pos	LPC 16:0 sn-2[+H]	496.339	14.44	Decreased***	**391**	Lipid-pos	PC 35:6[+H]	764.523	7.00	
**326**	LC-MS-pos	LPC-O 16:0[+H]	482.36	14.80		**392**	Lipid-pos	PC 36:0[+H]	790.632	9.30	Decreased**
**327**	Lipid-pos	LPC 16:1[+H]	494.325	1.54	Decreased*	**393**	Lipid-pos	PC 36:1[+H]	788.617	8.78	Decreased***
**328**	LC-MS-pos	LPC 17:0 sn-1[+H]	510.355	15.18	Decreased***	**394**	Lipid-pos	PC 36:2[+H]	786.602	8.12	Decreased***
**329**	LC-MS-pos	LPC 17:0 sn-2[+H]	510.355	16.00	Decreased***	**395**	Lipid-pos	PC 36:3[+H]	784.586	7.50	Decreased**
**330**	LC-MS-pos	LPC-O 18:1[+H]	508.378	15.28		**396**	Lipid-pos	PC 36:4[+H]	782.568	7.88	Decreased***
**331**	Lipid-pos	LPC-O 18:0[+H]	510.393	2.67		**397**	Lipid-pos	PC 36:5[+H]	780.555	6.62	
**332**	LC-MS-pos	LPC-O 18:2[+H]	506.361	15.36	Decreased***	**398**	Lipid-pos	PC(16:0_20:5)[+H]	780.553	7.22	Decreased**
**333**	LC-MS-pos	LPC 18:1 sn-1[+H]	522.355	14.71	Decreased***	**399**	Lipid-pos	PC 36:6[+H]	778.54	5.92	
**334**	LC-MS-pos	LPC 18:1 sn-2[+H]	522.355	14.95	Decreased***	**400**	Lipid-pos	PC 37:3[+H]	798.601	9.34	

(continued)

ID	Platform	Compounds[adduct ion]	m/z	t_R(min)	Gliomas
401	Lipid-pos	PC 37:4[+H]	796.586	7.64	Decreased***
402	Lipid-pos	PC 37:6[+H]	792.555	6.46	Decreased*
403	Lipid-pos	PC 37:7[+H]	790.538	7.21	Decreased**
404	Lipid-pos	PC 38:1[+H]	816.649	9.32	Decreased***
405	Lipid-pos	PC 38:3[+H]	812.618	8.34	Decreased**
406	Lipid-pos	PC 38:4[+H]	810.601	7.73	Decreased**
407	Lipid-pos	PC 38:5[+H]	808.586	7.15	
408	Lipid-pos	PC(16:0_22:5)[+H]	808.583	8.10	Decreased***
409	Lipid-pos	PC 38:6[+H]	806.57	6.97	Decreased**
410	Lipid-pos	PC 38:7[+H]	804.555	6.16	
411	Lipid-pos	PC 39:4[+H]	824.617	9.30	Decreased***
412	Lipid-pos	PC 39:5[+H]	822.602	9.07	
413	Lipid-pos	PC 39:6[+H]	820.586	7.40	Decreased***
414	Lipid-pos	PC 40:0[+H]	846.696	10.71	Decreased*
415	Lipid-pos	PC 40:1[+H]	844.68	9.99	Decreased**
416	Lipid-pos	PC 40:2[+H]	842.665	9.43	Decreased**
417	Lipid-pos	PC 40:3[+H]	840.649	8.85	Decreased**
418	Lipid-pos	PC 40:4[+H]	838.633	8.58	Decreased**
419	Lipid-pos	PC 40:5[+H]	836.617	7.74	Decreased*
420	Lipid-pos	PC 40:6[+H]	834.601	7.77	Decreased**
421	Lipid-pos	PC(16:0_24:6)[+H]	834.601	7.26	
422	Lipid-pos	PC 40:7[+H]	832.586	7.03	Decreased**
423	Lipid-pos	PC 41:1[+H]	858.696	10.34	Decreased***
424	Lipid-pos	PC 42:1[+H]	872.712	10.61	Decreased**
425	Lipid-pos	PC 42:10[+H]	854.571	6.25	Decreased**
426	Lipid-pos	PC 42:11[+H]	852.552	6.53	
427	Lipid-pos	PC 42:2[+H]	870.695	10.23	Decreased***
428	Lipid-pos	PC 42:4[+H]	866.665	9.12	Decreased***
429	Lipid-pos	PC 42:5[+H]	864.648	8.53	Decreased**
430	Lipid-pos	PC 42:7[+H]	860.615	7.87	Decreased**
431	Lipid-pos	PC 42:9[+H]	856.586	6.63	Decreased**
432	Lipid-pos	PC 44:2[+H]	898.728	10.70	Decreased***
433	Lipid-pos	PC 44:4[+H]	894.696	10.32	Decreased**
434	Lipid-pos	PC 44:5[+H]	892.681	9.66	Decreased***
435	Lipid-pos	PC-O 32:0[+H]	720.591	8.30	
436	Lipid-pos	PC-O 32:1[+H]	718.576	7.75	Decreased**
437	Lipid-pos	PC-O 32:2 (18:1)[+H]	716.56	7.57	Decreased**
438	Lipid-pos	PC-O 32:2[+H]	716.559	7.68	
439	Lipid-pos	PC-O 33:0[+H]	734.607	8.68	Decreased**
440	Lipid-pos	PC-O 34:1[+H]	746.607	8.38	Decreased***
441	Lipid-pos	PC-O 34:2[+H]	744.59	7.78	Decreased**
442	Lipid-pos	PC-O 34:2(18:0)[+H]	744.592	8.37	Decreased***
443	Lipid-pos	PC-O 35:2[+H]	758.607	8.84	Decreased***
444	Lipid-pos	PC-O 36:1[+H]	774.639	9.15	Decreased***
445	Lipid-pos	PC-O 36:2[+H]	772.622	8.57	Decreased***
446	Lipid-pos	PC-O 36:3[+H]	770.606	8.03	
447	Lipid-pos	PC-O 36:4[+H]	768.591	7.75	
448	Lipid-pos	PC-O 36:5[+H]	766.576	7.63	
449	Lipid-pos	PC-O 38:2[+H]	800.654	9.25	Decreased**
450	Lipid-pos	PC-O 38:3[+H]	798.639	8.94	
451	Lipid-pos	PC-O 38:4[+H]	796.623	8.60	
452	Lipid-pos	PC-O 38:5[+H]	794.606	7.89	
453	Lipid-pos	PC-O 38:6[+H]	792.591	7.41	
454	Lipid-pos	PC-O 38:7[+H]	790.573	7.81	
455	Lipid-pos	PC-O 38:7(22:5)[+H]	790.573	7.78	
456	Lipid-pos	PC-O 38:8[+H]	788.557	7.72	
457	Lipid-pos	PC-O 40:3[+H]	824.655	8.85	Decreased***
458	Lipid-pos	PC-O 40:5[+H]	822.638	8.81	Decreased**
459	Lipid-pos	PC-O 40:6[+H]	820.623	8.30	Decreased**
460	Lipid-pos	PC-O 40:7[+H]	818.607	7.55	
461	Lipid-pos	PC-O 44:6[+H]	876.686	9.48	
462	Lipid-neg	PE(14:0_20:4)[-H]	710.474	6.44	
463	Lipid-neg	PE(14:0_22:6)[-H]	734.474	6.10	
464	Lipid-neg	PE(15:0_18:1)[-H]	702.505	7.67	Decreased**
465	Lipid-neg	PE(16:0_16:1)[-H]	688.489	7.38	
466	Lipid-neg	PE(16:0_18:1)[-H]	716.521	8.00	
467	Lipid-neg	PE(16:0_20:4)[-H]	738.505	7.32	
468	Lipid-neg	PE(16:0_22:5)[-H]	764.522	7.29	
469	Lipid-neg	PE(16:0_22:6)[-H]	762.505	7.09	
470	Lipid-neg	PE(16:0p_16:0)[-H]	674.511	8.44	
471	Lipid-neg	PE(16:0p_16:1)[-H]	672.495	7.79	Decreased**
472	Lipid-neg	PE(16:0p_17:1)[-H]	686.511	8.15	Decreased***
473	Lipid-neg	PE(16:0p_18:1)[-H]	700.527	8.62	Decreased***
474	Lipid-neg	PE(16:0p_20:1)[-H]	728.558	9.23	Decreased***
475	Lipid-neg	PE(16:0p_20:3)[-H]	724.526	8.15	Decreased**
476	Lipid-neg	PE(16:0p_20:4)[-H]	722.51	7.85	
477	Lipid-neg	PE(16:0p_22:1)[-H]	756.589	9.86	Decreased***
478	Lipid-neg	PE(16:0p_22:4)[-H]	750.542	8.42	Decreased**
479	Lipid-neg	PE(16:0p_22:5)[-H]	748.526	7.78	
480	Lipid-neg	PE(16:0p_22:6)[-H]	746.511	7.55	
481	Lipid-neg	PE(16:1_18:1)[-H]	714.505	7.40	Decreased*
482	Lipid-neg	PE(16:1_20:4)[-H]	736.49	6.60	
483	Lipid-neg	PE(16:1_22:6)[-H]	760.489	6.30	
484	Lipid-neg	PE(16:1p_20:4)[-H]	720.495	7.06	
485	Lipid-neg	PE(17:0_18:1)[-H]	730.538	8.45	Decreased***
486	Lipid-neg	PE(17:0_20:4)[-H]	752.521	7.80	Decreased**
487	Lipid-neg	PE(17:0_22:6)[-H]	776.521	7.47	Decreased**
488	Lipid-neg	PE(17:1_18:1)[-H]	728.521	7.87	Decreased***
489	Lipid-neg	PE(17:1_20:4)[-H]	750.505	7.08	Decreased***
490	Lipid-neg	PE(18:0_16:1)[-H]	716.521	8.19	
491	Lipid-neg	PE(18:0_18:1)[-H]	744.553	8.83	
492	Lipid-neg	PE(18:0_20:1)[-H]	772.583	9.48	Decreased***
493	Lipid-neg	PE(18:0_20:2)[-H]	770.568	9.03	Decreased**
494	Lipid-neg	PE(18:0_20:3)[-H]	768.552	8.82	
495	Lipid-neg	PE(18:0_20:4)[-H]	766.537	8.21	
496	Lipid-neg	PE(18:0_22:3)[-H]	796.584	9.27	
497	Lipid-neg	PE(18:0_22:5)[-H]	792.553	8.52	
498	Lipid-neg	PE(18:0_22:6)[-H]	790.536	7.97	
499	Lipid-neg	PE(18:0p_16:1)[-H]	700.526	8.79	
500	Lipid-neg	PE(18:0p_18:1)[-H]	728.558	9.37	Decreased***
501	Lipid-neg	PE(18:0p_20:3)[-H]	752.558	9.21	
502	Lipid-neg	PE(18:0p_20:4)[-H]	750.542	8.68	
503	Lipid-neg	PE(18:0p_22:4)[-H]	778.573	9.13	Decreased**
504	Lipid-neg	PE(18:0p_22:5)[-H]	776.557	8.97	
505	Lipid-neg	PE(18:0p_22:6)[-H]	774.542	8.36	
506	Lipid-neg	PE(18:0p_24:2)[-H]	810.636	10.55	Decreased**
507	Lipid-neg	PE(18:1_18:1)[-H]	742.537	8.23	Decreased*
508	Lipid-neg	PE(18:1_18:2)[-H]	740.521	7.58	Decreased**
509	Lipid-neg	PE(18:1_18:3)[-H]	738.505	7.10	
510	Lipid-neg	PE(18:1_20:2)[-H]	768.553	8.50	
511	Lipid-neg	PE(18:1_20:3)[-H]	766.536	7.81	Decreased*
512	Lipid-neg	PE(18:1_20:4)[-H]	764.521	7.44	
513	Lipid-neg	PE(18:1_22:0)[-H]	800.615	10.29	
514	Lipid-neg	PE(18:1_22:1)[-H]	798.599	9.63	
515	Lipid-neg	PE(18:1_24:0)[-H]	828.646	10.88	
516	Lipid-neg	PE(18:1p_18:1)[-H]	726.542	8.67	Decreased***
517	Lipid-neg	PE(18:1p_20:1)[-H]	754.573	9.39	Decreased***
518	Lipid-neg	PE(18:1p_20:2)[-H]	752.557	8.85	Decreased***
519	Lipid-neg	PE(18:1p_20:4)[-H]	748.526	7.96	Decreased***
520	Lipid-neg	PE(18:1p_21:3)[-H]	764.558	8.77	Decreased***
521	Lipid-neg	PE(18:1p_22:1)[-H]	782.604	9.90	Decreased**
522	Lipid-neg	PE(18:1p_22:5)[-H]	774.541	8.18	
523	Lipid-neg	PE(18:1p_22:6)[-H]	772.526	7.68	Decreased**
524	Lipid-neg	PE(18:2_20:4)[-H]	762.505	6.79	Decreased*
525	Lipid-neg	PE(18:2_22:6)[-H]	786.506	6.47	
526	Lipid-neg	PE(18:2p_20:4)[-H]	746.51	7.29	Decreased*
527	Lipid-neg	PE(20:0_20:4)[-H]	794.568	9.01	
528	Lipid-neg	PE(20:0p_20:4)[-H]	778.573	9.45	
529	Lipid-neg	PE(20:0p_22:6)[-H]	802.572	9.14	
530	Lipid-neg	PE(20:1p_22:4)[-H]	804.589	9.15	Decreased***
531	Lipid-neg	PE(20:1p_22:6)[-H]	800.557	8.45	Decreased**
532	Lipid-neg	PE(22:0_20:4)[-H]	822.599	9.73	
533	Lipid-neg	PE(22:3_20:4)[-H]	816.552	8.00	
534	Lipid-pos	PG(16:0_18:1)[+NH4]	766.56	6.56	

(continued)

ID	Platform	Compounds[adduct ion]	m/z	t_R(min)	Gliomas	ID	Platform	Compounds[adduct ion]	m/z	t_R(min)	Gliomas
**535**	Lipid-pos	PG(16:0_20:4)[+NH4]	788.545	5.92		**602**	Lipid-pos	DG(18:0_20:4)[+NH4]	662.573	9.30	
**536**	Lipid-pos	PG(18:1_18:1)[+NH4]	792.576	6.15	Decreased***	**603**	Lipid-pos	DG(18:0_22:4)[+NH4]	690.604	9.78	Decreased*
**537**	Lipid-pos	PG(18:1_18:2)[+NH4]	790.56	5.48	Decreased***	**604**	Lipid-pos	DG(18:0_22:6)[+NH4]	686.573	9.08	
**538**	Lipid-pos	PG(18:1_20:4)[+NH4]	814.561	5.46	Decreased***	**605**	Lipid-pos	DG(18:1_18:1)[+NH4]	638.573	9.40	Decreased**
**539**	Lipid-pos	PG(18:2_18:2)[+NH4]	788.545	4.84		**606**	Lipid-pos	DG(18:1_18:2)[+NH4]	636.557	8.81	
**540**	Lipid-pos	PG(18:2_22:6)[+NH4]	836.545	4.54		**607**	Lipid-pos	DG(18:1_20:4)[+NH4]	660.557	8.65	
**541**	Lipid-pos	PG 34:2[+NH4]	764.545	5.94		**608**	Lipid-pos	DG(18:1_22:4)[+NH4]	688.589	9.20	
**542**	Lipid-neg	PI(16:0_20:3)[-H]	859.531	5.83	Decreased*	**609**	Lipid-pos	DG(18:1_22:6)[+NH4]	684.557	8.43	
**543**	Lipid-neg	PI(16:1_18:1)[-H]	833.515	5.79	Decreased***	**610**	Lipid-pos	TG(10:0_16:0_16:0)[+NH4]	740.678	11.81	
**544**	Lipid-neg	PI(16:1_20:4)[-H]	855.502	5.01	Decreased***	**611**	Lipid-pos	TG(10:0_16:0_18:1)[+NH4]	766.693	11.88	
**545**	Lipid-neg	PI(17:0_20:4)[-H]	871.531	6.31	Decreased***	**612**	Lipid-pos	TG(10:0_18:1_18:3)[+H]	771.648	11.89	
**546**	Lipid-neg	PI(18:0_20:3)[-H]	887.562	6.97	Decreased**	**613**	Lipid-pos	TG(14:0_20:4_20:4)[+H]	875.711	12.24	
**547**	Lipid-neg	PI(18:0_20:4)[-H]	885.547	6.73	Decreased***	**614**	Lipid-pos	TG(15:0_18:1_18:2)[+NH4]	860.772	12.58	
**548**	Lipid-neg	PI(18:0_22:6)[-H]	909.547	6.45	Decreased**	**615**	Lipid-pos	TG(16:0_12:0_18:1)[+NH4]	794.724	12.26	
**549**	Lipid-neg	PI(18:1_20:4)[-H]	883.531	6.04		**616**	Lipid-pos	TG(16:0_12:0_18:3)[+H]	773.664	12.22	
**550**	Lipid-neg	PI-O 36:4 (20:4)[-H]	843.536	6.15	Decreased**	**617**	Lipid-pos	TG(16:0_12:0_20:4)[+NH4]	816.709	11.79	
**551**	Lipid-neg	PI-O 38:5 (20:4)[-H]	869.552	6.47	Decreased***	**618**	Lipid-pos	TG(16:0_14:0_14:0)[+NH4]	768.709	12.22	
**552**	Lipid-pos	PS(16:0_18:1)[+H]	762.529	6.50		**619**	Lipid-pos	TG(16:0_14:0_16:0)[+NH4]	796.74	12.60	
**553**	Lipid-pos	PS(16:0_20:4)[+H]	784.514	5.77		**620**	Lipid-pos	TG(16:0_14:0_20:5)[+H]	825.696	12.30	
**554**	Lipid-pos	PS(16:0_22:6)[+H]	808.514	5.47		**621**	Lipid-pos	TG(16:0_15:0_16:0)[+NH4]	810.755	12.81	
**555**	Lipid-neg	PS(18:0_18:1)[-H]	788.542	7.60	Decreased***	**622**	Lipid-pos	TG(16:0_15:0_16:1)[+NH4]	808.739	12.50	
**556**	Lipid-neg	PS(18:0_20:2)[-H]	814.557	7.50	Decreased**	**623**	Lipid-pos	TG(16:0_15:0_18:1)[+NH4]	836.771	12.85	
**557**	Lipid-pos	PS(18:0_20:3)[+H]	814.56	6.95	Decreased**	**624**	Lipid-pos	TG(16:0_15:0_18:2)[+NH4]	834.755	12.54	
**558**	Lipid-neg	PS(18:0_22:4)[-H]	838.557	7.31	Decreased**	**625**	Lipid-pos	TG(16:0_16:0_16:0)[+NH4]	824.771	12.97	
**559**	Lipid-pos	PS(18:0_22:5)[+H]	838.56	7.07		**626**	Lipid-pos	TG(16:0_16:0_16:1)[+NH4]	822.755	12.74	
**560**	Lipid-pos	PS(18:0_22:6)[+H]	836.544	6.45		**627**	Lipid-pos	TG(16:0_16:0_16:2)[+NH4]	820.739	12.32	
**561**	Lipid-pos	PS(18:1_18:1)[+H]	788.545	6.68	Decreased***	**628**	Lipid-pos	TG(16:0_16:0_17:0)[+NH4]	838.787	13.21	
**562**	Lipid-pos	PS(18:1_18:2)[+H]	786.529	5.99	Decreased**	**629**	Lipid-pos	TG(16:0_16:0_20:4)[+NH4]	872.771	12.64	
**563**	Lipid-pos	PS(18:1_20:3)[+H]	812.545	6.25	Decreased**	**630**	Lipid-pos	TG(16:0_16:0_22:6)[+NH4]	896.771	12.50	
**564**	Lipid-pos	PS(18:1_22:6)[+H]	834.53	5.76	Decreased**	**631**	Lipid-pos	TG(16:0_16:1_18:1)[+NH4]	848.771	12.78	
**565**	Lipid-neg	PS(18:1_24:6)[-H]	860.541	6.12	Decreased*	**632**	Lipid-pos	TG(16:0_16:1_18:2)[+NH4]	846.755	12.41	
**566**	Lipid-pos	PS(20:3_22:6)[+H]	858.529	5.51	Decreased**	**633**	Lipid-pos	TG(16:0_17:0_18:1)[+NH4]	864.803	13.25	
**567**	Lipid-pos	PS(22:3_22:6)[+H]	886.56	6.21		**634**	Lipid-pos	TG(16:0_17:1_18:1)[+NH4]	862.787	12.92	
**568**	Lipid-pos	PS(22:4_22:6)[+H]	884.545	5.45		**635**	Lipid-pos	TG(16:0_18:1_18:1)[+NH4]	876.803	13.03	
**569**	Lipid-neg	PS-O 39:7 (22:6)[-H]	790.5	5.88		**636**	Lipid-pos	TG(16:0_18:1_18:2)[+NH4]	874.787	12.82	
**570**	Lipid-pos	SM(d16:0/15:1)[+H]	661.529	5.49		**637**	Lipid-pos	TG(16:0_18:1_20:4)[+NH4]	898.787	12.68	
**571**	Lipid-pos	SM(d16:0/16:0)[+H]	677.561	6.30		**638**	Lipid-pos	TG(16:0_18:1_22:6)[+NH4]	922.787	12.54	
**572**	Lipid-pos	SM(d16:0/16:1)[+H]	675.545	5.95		**639**	Lipid-pos	TG(16:0_18:2_18:2)[+NH4]	872.771	12.45	
**573**	Lipid-pos	SM(d16:0/17:1)[+H]	689.56	6.44		**640**	Lipid-pos	TG(16:0_18:2_18:3)[+H]	853.726	12.68	
**574**	Lipid-pos	SM(d16:0/18:0)[+H]	705.591	7.21	Elevated **	**641**	Lipid-pos	TG(16:0_18:2_20:4)[+NH4]	896.772	12.31	
**575**	Lipid-pos	SM(d16:0/18:1)[+H]	703.576	6.90		**642**	Lipid-pos	TG(16:0_20:3_20:3)[+H]	907.774	13.24	
**576**	Lipid-pos	SM(d16:0/18:2)[+H]	701.56	6.11		**643**	Lipid-pos	TG(16:0_20:3_22:6)[+H]	929.758	12.83	
**577**	Lipid-pos	SM(d16:0/19:1)[+H]	717.591	7.41	Decreased*	**644**	Lipid-pos	TG(17:0_18:1_18:2)[+NH4]	888.803	13.01	
**578**	Lipid-pos	SM(d16:0/20:0)[+H]	733.623	8.14	Elevated *	**645**	Lipid-pos	TG(17:0_18:1_20:5)[+H]	893.758	12.97	
**579**	Lipid-pos	SM(d16:0/20:1)[+H]	731.607	7.83		**646**	Lipid-pos	TG(17:1_18:1_18:2)[+NH4]	886.787	12.63	
**580**	Lipid-pos	SM(d16:0/20:3)[+H]	727.574	7.24		**647**	Lipid-pos	TG(17:1_18:2_18:3)[+H]	865.727	12.60	
**581**	Lipid-pos	SM(d16:0/20:4)[+H]	725.558	6.90		**648**	Lipid-pos	TG(18:0_16:0_16:0)[+NH4]	852.803	13.37	
**582**	Lipid-pos	SM(d16:0/20:5)[+H]	723.542	6.14		**649**	Lipid-pos	TG(18:0_16:0_16:1)[+NH4]	850.787	13.04	
**583**	Lipid-pos	SM(d16:0/22:1)[+H]	759.638	8.66		**650**	Lipid-pos	TG(18:0_16:0_20:4)[+H]	883.774	13.44	
**584**	Lipid-pos	SM(d16:0/22:3)[+H]	755.604	8.17	Elevated **	**651**	Lipid-pos	TG(18:0_16:0_22:4)[+NH4]	928.835	13.26	
**585**	Lipid-pos	SM(d16:0/22:4)[+H]	753.589	7.85		**652**	Lipid-pos	TG(18:0_18:1_18:2)[+NH4]	902.819	13.14	
**586**	Lipid-pos	SM(d16:0/22:5)[+H]	751.573	7.11	Decreased**	**653**	Lipid-pos	TG(18:0_18:1_20:4)[+NH4]	926.818	13.08	
**587**	Lipid-pos	SM(d16:0/24:1)[+H]	787.67	9.52	Elevated ***	**654**	Lipid-pos	TG(18:0_18:2_22:6)[+NH4]	948.803	12.56	
**588**	Lipid-pos	SM(d16:0/24:2)[+H]	785.655	8.96		**655**	Lipid-pos	TG(18:0_20:3_22:6)[+NH4]	974.818	12.62	
**589**	Lipid-pos	SM(d16:0/26:1)[+H]	815.701	9.71	Decreased**	**656**	Lipid-pos	TG(18:0_20:4_22:5)[+NH4]	974.818	12.81	
**590**	Lipid-pos	SM(d16:1/16:1)[+H]	673.53	5.11	Decreased*	**657**	Lipid-pos	TG(18:0_20:4_22:6)[+NH4]	972.802	12.43	
**591**	Lipid-pos	SM(d18:1/17:1)[+H]	715.575	6.64	Decreased**	**658**	Lipid-pos	TG(18:1_18:1_18:2)[+NH4]	900.803	12.86	
**592**	Lipid-pos	SM(d22:0/19:1)[+H]	801.686	9.90		**659**	Lipid-pos	TG(18:1_18:1_18:3)[+H]	881.758	13.10	
**593**	Lipid-pos	SM(d22:1/19:1)[+H]	799.67	9.35		**660**	Lipid-pos	TG(18:1_18:1_20:3)[+NH4]	926.819	12.90	
**594**	Lipid-pos	SM(d24:0/19:1)[+H]	829.717	10.63		**661**	Lipid-pos	TG(18:1_18:1_20:4)[+NH4]	924.803	12.72	
**595**	Lipid-pos	DG(16:0_16:0)[+NH4]	586.542	9.16		**662**	Lipid-pos	TG(18:1_18:1_20:5)[+H]	905.758	12.79	
**596**	Lipid-pos	DG(16:0_18:1)[+NH4]	612.557	9.26	Decreased**	**663**	Lipid-pos	TG(18:1_18:2_18:2)[+NH4]	898.787	12.49	
**597**	Lipid-pos	DG(16:0_20:4)[+NH4]	634.541	8.53		**664**	Lipid-pos	TG(18:1_18:2_20:5)[+H]	903.742	12.51	
**598**	Lipid-pos	DG(16:0_22:4)[+NH4]	662.573	9.09		**665**	Lipid-pos	TG(18:2_18:2_18:2)[+NH4]	896.772	12.13	
**599**	Lipid-pos	DG(16:0_22:6)[+NH4]	658.541	8.27		**666**	Lipid-pos	TG(20:3_20:3_20:4)[+H]	955.773	12.82	
**600**	Lipid-pos	DG(18:0_16:0)[+NH4]	614.573	9.87		**667**	Lipid-pos	TG(20:3_20:4_22:6)[+H]	977.758	12.26	
**601**	Lipid-pos	DG(18:0_20:3)[+NH4]	664.589	9.60	Decreased*	**668**	Lipid-pos	TG(20:5_20:4_22:6)[+H]	973.728	11.81	
